# Evaluating a decision making system for cardiovascular dysautonomias diagnosis

**DOI:** 10.1186/s40064-016-1730-7

**Published:** 2016-01-26

**Authors:** Ali Idri, Ilham Kadi

**Affiliations:** Software Project Management Research Team, ENSIAS, Mohammed V University in Rabat, Rabat, Morocco

**Keywords:** Cardiovascular dysautonomias, Data mining, C4.5 algorithm, Decision tree

## Abstract

Autonomic nervous system (ANS) is the part of the nervous system that is involved in homeostasis of the whole body functions. A malfunction in this system can lead to a cardiovascular dysautonomias. Hence, a set of dynamic tests are adopted in ANS units to diagnose and treat patients with cardiovascular dysautonomias. The purpose of this study is to develop and evaluate a decision tree based cardiovascular dysautonomias prediction system on a dataset collected from the ANS unit of the Moroccan university hospital Avicenne. We collected a dataset of 263 records from the ANS unit of the Avicenne hospital. This dataset was split into three subsets: training set (123 records), test set (55 records) and validation set (85 records). C4.5 decision tree algorithm was used in this study to develop the prediction system. Moreover, Java Enterprise Edition platform was used to implement a prototype of the developed system which was deployed in the Avicenne ANS unit so as to be clinically validated. The performance of the decision tree-based prediction system was evaluated by means of the error rate criterion. The error rates were measured for each classifier and have achieved an average value of 1.46, 2.24 and 0.89 % in training, test, and validation sets respectively. The results obtained were encouraging but further replicated studies are still needed to be performed in order to confirm the findings of this study.

## Introduction

Data mining (DM) is an analytic process designed to explore large datasets in hidden and previously unknown patterns, relationships and knowledge. According to Fayyad et al. (1996), data mining is defined as “a process of nontrivial extraction of implicit, previously unknown and potentially useful information from the data stored in a database”. Thereby, data mining can be considered as knowledge discovery from data including three phases namely data pre-processing, data modeling and data post-processing (Fayyad et al. 1996). Recent researches have shown that applications of data mining in several fields such as education (Romero and Ventura 2010, Romero et al. 2008), clinical medicine (Bellazzi and Zupan 2008) and financial fraud detection (Kirkos et al. 2007) are growing. Classification is one of the main tasks of DM. In fact, classification techniques are capable of processing a large amount of data. They may predict categorical class labels and classify data based on a training set (Aparna et al. 2012). Decision trees (DT) algorithms are considered as one of the most popular classification techniques. The goal of DTs is creating a model that predicts the value of a target variable (class) by learning simple decision rules inferred from the data features. Indeed, decision tree algorithms break down a dataset into smaller and smaller subsets while at the same time an associated decision tree is incrementally developed. The final result is a tree with decision nodes and leaf nodes (Apté and Weiss 1997). There are many specific decision-tree algorithms. Notable ones include: ID3, C4.5 (Quinlan 1993), and CART (Breiman et al. 1984).

C4.5 algorithm is one of the well-known decision tree algorithms because of its efficiency and comprehensive features (Quinlan 1993). It is an improved extension of ID3 algorithm and it has been used in different disciplines of medical field including cardiology (Esfandiari et al. 2014). Pavlopoulos et al. (Pavlopoulos et al. 2004) used the C4.5 algorithm to analyze different heart sound features, which assist clinicians to make a better diagnosis in Coronary Heart Disease (CHD). Mašetić and Subasi have evaluated the effect of C4.5 decision tree in creating a model that will detect and separate normal and Congestive Heart Failures (CHF) on the long-term ECG time series. Experimental results showed that C4.5 algorithm has significant role in identification and classification of ECG (ElectroCardioGram) heartbeat signals with an accuracy of 99.86 % (Mašetic and Subasi 2013). Karaolis et al. developed a decision tree based system for the assessment of coronary heart disease related risk factors targeting in the reduction of CHD events. The system has shown highest and promising accuracy rates (Karaolis et al. 2010). Overall, the results obtained by studies applying C4.5 algorithm in cardiology were satisfactory. However, to the best of our knowledge, there is no existing study that applies C4.5 algorithm in the ANS field.

Autonomic nervous system (ANS) is the designation applied by John Langley (Langley 1921) to a complex network of peripheral nerves and ganglia. It is the part of the nervous system that is involved in homeostasis by coordinating internal functions of the body and regulating automatically different organs including the cardiovascular system. ANS is the motor time (innervations of smooth muscle fibers) and sensory (pain in tension, compression, repletion) (Kreibig 2010). However, ANS is frequently subject to malfunctions that may cause serious problems and can be, in some cases, life-threatening. This is why, a set of dynamic tests including deep breathing, mental stress, hand-grip, Valsalva maneuver and tilt tests were identified to allow the diagnosis of autonomic disorders. In fact, autonomic testing is designed to determine how well the body is regulating the blood pressure (BP) and heart rate (HR). During these tests, the HR and BP are measured continuously. The tests include asking the patient to breathe deeply for 2 min, breathing as fast and as hard as he can for 30 s, maintaining a handgrip for 3 min, breathing against pressure for 15 s and observes if standing up leads to a sudden fall in blood pressure and/or an excessive increase in pulse rate or fainting. All these tests are meant to stimulate the ANS to produce changes in BP and HR of short duration which may reflect how well the involuntary nervous system is working.

In this paper, an experiment was performed in the ANS unit of the Moroccan university hospital Avicenne. This unit is specialized in diagnosing patients with cardiovascular dysautonomias by performing several dynamic tests and providing them appropriate treatments. This process includes two major stages: in the first stage, the test results are analyzed to deduce the preliminary conclusions for each ANS test; and in the second stage, these preliminary conclusions are analyzed taking into consideration other attributes to conclude the final diagnosis. In this paper, we only focus on the first stage of the ANS procedure to automate the generation of the preliminary conclusions. In fact, the analysis process of the test results carried out in this unit was done until now manually by the specialists. This method may be challenging and time consuming, especially with the growing number of patients at the same time. Thus, in order to help those ANS specialists, we were motivated to perform this study that aims at building a C4.5 decision tree based cardiovascular dysautonomias prediction system. The proposed ANS prediction system was evaluated on a dataset collected from the ANS unit of Avicenne hospital.

The article is organized as follow: “Background” section presents the ANS concepts as well as the C4.5 algorithm. “Methods” section presents a description of the medical dataset used as well as of the experimental design. Fourth section presents and discusses the results obtained. Finally, the conclusion and future work are presented in fifth section.

## Background

In this section, an overview of the ANS is presented. Thereafter, a brief presentation of C4.5 decision tree algorithm is introduced.

### Diagnosis tests of autonomic disorders

Autonomic nervous system is a complex network of peripheral nerves and ganglia. This system is involved in homeostasis by coordinating internal functions of the body and regulating automatically certain body processes. It is the part of the nervous system that controls involuntary actions, such as the beating of the heart and the widening or narrowing of the blood vessels. It controls, in particular, smooth muscle, heart muscle, some endocrine glands and the majority of exocrine glands (digestion, sweating, etc.). Thus, the ANS system controls blood pressure, heart and breathing rates, body temperature, digestion, metabolism (thus affecting body weight), the production of body fluids (saliva, sweat, and tears), and other processes (Kreibig 2010).

The ANS is divided into two complementary systems: the sympathetic nervous system (SNS) and the parasympathetic nervous system (PNS). The balance of these two systems provides the balance of physiological functions (Benarroch 1993). The sympathetic division prepares the body for stressful or emergency situations. It accelerates the heart rate, constricts blood vessels, and raises blood pressure. Besides, it causes the body to release stored energy and increases the muscular strength. The PNS part controls body process during ordinary situations. Generally, it conserves and restores the energy. It slows the heart rate, decreases blood pressure, increases intestinal and gland activity, and relaxes sphincter muscles.

The ANS is frequently subject to malfunctions that are called dysautonomias. ANS disorders can occur alone or as a result of another disease, such as Parkinson disease, alcoholism and diabetes (Grubb and Karas 1999). These disorders can cause serious troubles related to blood pressure, heart, breathing, swallowing and others. When they affect the breathing and/or heart functions, these disorders can be life-threatening. Doctors can check for signs of autonomic disorders during the physical examination. They measure blood pressure and heart rate while a person is sitting and after the person stands.

In this paper, an experiment was conducted in the ANS unit of the Moroccan university hospital Avicenne. This unit is specialized on performing ANS tests to diagnose patients with cardiovascular dysautonomias and provides them appropriate treatments. For each test, the specialists analyze deeply the obtained results of HR and BP and provide an interpretation for each result. These interpretations are called preliminary conclusions. Hence, a set of preliminary conclusions is deduced in the first stage of the ANS procedure. Table 1 provides descriptions of HR and BP values extracted from the training set, maximum and minimum values of HR and BP in each ANS test were measured using the training set. As can be seen, Table 1 contains very low and very high values of HR and BP that are considered in cardiology very dangerous and can be life threatening. For this reason, the ANS specialists perform these tests and measure the HR and BP values for each patient in different positions to provide meaningful interpretations.

**Table 1:** Description and statistics of HR and BP values across the training set for each ANS test

ANS tests	Measured values	Heart rate		Blood pressure	
		Min	Max	Min	Max
Deep breathing	VR	38	128	–	–
Hand grip	VR	36	120	–	–
	PSR α	–	–	87	228
Mental stress test	CSR α	–	–	74	203
	CSR β	36	129	–	–
Orthostatic test	VR	39	144	–	–
	SP_HR	18	165	–	–
	SP_BP	–	–	85	139

The tests conducted by the ANS unit of the Moroccan university hospital Avicenne are:

- Deep breathing (DB) (Shields 2009): it has a major interest in the determination of the vagal response (VR). It assesses autonomic function by measuring changes in HR in response to a deep breath. The calculation of VR is obtained by means of Eq. 1.
  1 $${\hbox{VR}} = \frac{{HR_{\hbox{max} } - HR_{\hbox{min} } }}{{HR_{\hbox{min} } }} \times 100$$
- Hand grip (HG) (Johansen et al. 1997): This is a manual effort contraction performed to determine changes in BP in static effort. In normal conditions, a muscle contraction increases HR and BP. In this test, two values are measured: VR, by the same method as Deep breathing test, and Peripheral sympathetic alpha (PSR) activity by means of Eq. 2.
  2 $${\text{PSR}}\,\upalpha = \frac{{BP_{\hbox{max} } - BP_{\hbox{min} } }}{{BP_{\hbox{min} } }} \times 100$$
- Mental stress (MS) (Johansen et al. 1997): The patient performs mental arithmetic calculations. The result is an increase in BP and HR by activating of the central sympathetic nerve (Low 1997). In mental stress, the central sympathetic nerves activities “α” is evaluated by measuring the variations of BP using Eq. 3:
  3 $${\text{CSR}}\,\upalpha = \frac{{BP_{\hbox{max} } - BP_{\hbox{min} } }}{{BP_{\hbox{min} } }} \times 100$$
  The central sympathetic nerves activities “β” is evaluated by measuring the variations of HR using Eq. 4:
  4 $${\text{CSR}}\,\upbeta = \frac{{HR_{\hbox{max} } - HR_{\hbox{min} } }}{{HR_{\hbox{min} } }} \times 100$$
- Orthostatic test (Ort) (Mejía-Rodríguez et al. 2009): it aims at measuring HR and BP variations in different positions: stand up and rest. In fact, the transition from rest position to a standing position causes a variation in HR and BP and a variety of physiological processes of adaptation in normal subjects. Thereby, the specialists record several values of HR and BP during the orthostatic test in order to measure VR, HR and BP values in supine position.

### C4.5 decision tree algorithm: an overview

C4.5 is an algorithm for inducing classification rules in the form of a decision tree from data. It was introduced by Quinlan (Quinlan 1993) as an improvement of the basic ID3 algorithm. Such improvements consist on:

- Choosing an appropriate attribute selection measure,
- Handling missing data,
- Pruning the decision tree, and
- Handling numerical attributes.

C4.5 algorithm builds a decision tree from a set of training data using the concept of information entropy. It uses the divide-and-conquer approach to induce the decision tree. The algorithm uses a selected criterion to build the tree. It works top–down, seeking at each stage an attribute to best splitting the predefined classes (Han and Kamber 2001). C4.5 includes a post-pruning process to avoid overfitting. This process takes place after the induction of the entire tree. In fact, after a decision tree is produced, C4.5 prunes it in a single bottom-up pass. In addition, C4.5 algorithm uses heuristics for pruning the trees. Next, brief descriptions of the C4.5 as well as its pruning strategy used in this study are presented (Quinlan 1993).

*C4.5 algorithm*

**Input**

1. Training dataset S which contains observations and their predefined classes.
2. Attribute list A: a set of candidate attributes.
3. Selected splitting criterion (Gain ratio).

#### **Output**

A decision tree.

**Method**

- Create a node N_d_.
- If all observations in the training set have the same class output value C, then return N_d_ as a leaf node labeled with C.
- If attribute list is empty, then return N_d_ as leaf node labeled with the most frequent class value in the observations of this node.
- Apply the selected splitting criterion (Gain ratio) to training dataset in order to find the “best” splitting attribute X.
- Label node N_d_ with X.
- Remove the splitting attribute X from the attribute list.
- **For** each value Xj of X.
  - D_j_ is the set of observations in training dataset for which X = Xj.
  - If a stopping criterion is true for D_j_, then attach a leaf node labeled with the frequent class value of D_j_ to node N_d_.
  - Else attach the node returned by a generate decision tree (Dj, attribute list, selected splitting criterion) to node N_d_.
- Return node N_d_.
- When the tree is complete, post-pruning process is applied
- **For** each sub-tree T
  - **If** T can be replaced with a leaf node N_d_ without decreasing accuracy. Then replace T with N_d_.
  - **Else** maintain T.

## Material and methods

The development of the prediction system of this study went through the following major phases: data collection, data pre-processing, modeling and evaluation. In this section, the tasks performed in each phase are presented and explained.

### Medical dataset collection and description

A total of 263 records were collected from the ANS unit of the cardiology department of Avicenne hospital. All these records are collected from patients suffering from cardiovascular dysautonomias. This dataset was split into three subsets: training subset (123 records), test subset (55 records) and validation subset (85 records). The training subset was used to build the decision trees and the test one was used to evaluate the generated classifiers while the validation subset was used to clinically validate the developed prediction system. The training and test subsets include records of patients who went to ANS unit in the period between January 2013 and May 2014 while the validation subset includes records from the period between July and November 2014. Moreover, the classes required to generate the decision trees were identified based on the empirical knowledge of the four specialists of the ANS unit including the head of the cardiology department. These four specialists perform a deep analysis of the results obtained in each ANS test to all patients in order to deduce the preliminary conclusions. In case of disagreement, the head of the department is responsible for taking the appropriate decision. According to the specialist’s guidelines, three classes were identified for each ANS test namely: *high*, *normal* and *low*. A class distribution for all the ANS tests was evaluated and has shown that 41 % of the data were identified as *high* class, 31 % as *normal* class and 28 % as *low* class.

On the other hand, the dataset contains a set of attributes; some provide general and administrative information about the patient such as: name of patient, file reference, date of consultation and the attending physician. These data do not affect the patient diagnosis which is why they were discarded. Only the attributes judged by the specialists to be relevant for the diagnosis of patients were selected. Hence, 11 attributes were selected in this first stage of the ANS procedure to generate the decision trees. Table 2 provides a brief description as well as some statistics such as mean, max, and the min values of each selected attribute values using the training subset. According to Table 2, the patients diagnosed by ANS units are from all generations including the children and the oldest persons. For the other attributes, the age of patients is considered by the ANS specialists as the main factor influencing the results interpretation. In fact, a normal value of an ANS test result differs according to age category.

**Table 2:** Description and statistics of selected attributes

Input attributes	Description	Mean	Min	Max
Age (years)	Age of the patient	41.4	7	83
VR_DB (beat/min)	Vagal response measured using HR values in DB test	45.73	4	155
VR_HG (beat/min)	Vagal response measured using HR values in HG test	19.39	0	66
PSR α (mmHg)	Peripheral sympathetic response α measured using BP values in HG test	21.75	2	72
CSR α (mmHg)	Central sympathetic response α measured using BP values in MS test	16.35	3	56
CSR β (beat/min)	Central sympathetic response β measured using HR values in MS test	18.93	1	95
VR_Ort (beat/min)	Vagal response measured using HR values in Ort test	21.24	1	80
HR_min_ (beat/min)	Minimum heart rate measured in Ort test	61.51	17	104
HR_max_ (beat/min)	Maximum heart rate measured in Ort test	70.48	38	165
BP_min_ (mmHg)	Minimum blood pressure measured in Ort test	114.47	84	161
BP_max_ (mmHg)	Maximum blood pressure measured in Ort test	125.94	89	181

As an example, for the VR_DB attribute, a normal value should be near to 30 % for adult patients (18 < Age < 60). However, for the elderly, a normal value is around 25 %; and for children and young patients, a normal value is close to 60 %. Also, the same principle applies to VR_HG, PSR α, CSR α, CSR β and VR_Ort attributes except that the critical values differ from one attribute to another. Nevertheless, we notice that the average value for VR_DB is 46.23 % which shows that a lot of patients suffer from difficulties in case of breathing efforts. In general, a normal HR value should be between 60 beats/min and 80 beats/min, and a normal value of BP should be between 100 and 140 for systolic values. These values were calculated using devices that measures HR and BP directly from patients through a set of sensors. These devices are adopted by the specialists of the ANS unit for several years. According to Table 2, the min and max values detected have far exceeded the normal ones for both HR and BP which shows that there are some patients that are suffering from serious problems that need to be treated urgently.

### Pre-processing

Data preprocessing is a very important step in a data mining process. It is a critical step which deals with the preparation and transformation of the initial data. In fact, analyzing data that has not been carefully screened can produce misleading and/or biased results. Therefore, the quality and representation of data is first and foremost before running an analysis (Han et al. 2011). An initial dataset can generally present several problems such as: missing values, noisy data and inconsistency (Witten and Frank 1999). For this reason, several methods were developed to solve these challenges and improve the data quality. These methods can be divided in (Familia et al. 1997):

- Data cleaning: Fill in missing values, smooth noisy data, identify or remove outliers, and resolve inconsistencies.
- Data integration: Integration of multiple databases, data cubes, or files.
- Data transformation: Normalization and aggregation.
- Data reduction: Obtains reduced representation in volume but produces the same or similar analytical results.

The database used in this study, contained a few missing values that did not exceed 4 %. Thus, a data cleaning process is required. After examining the nature of the missing values, we noticed that they concern the following measures: VR_DB, VR_HG, PSR α, CSR α, CSR β and VR_Ort. As aforementioned, these values are obtained using the mathematical formulas identified in “Diagnosis tests of autonomic disorders” section, the required calculations were performed in order to fill the missing values. Furthermore, several attributes contained numeric values including real numbers which were all transformed to integer values. This transformation was carried out by keeping just the integer part.

### Classifier modeling

The modeling phase is related to the discovery of relationships between various data in order to extract hidden patterns. As previously explained in “Diagnosis tests of autonomic disorders” section, a cardiovascular dysautonomias patient’s diagnosis is based on several preliminary conclusions of ANS’s tests. For each test, one or more preliminary conclusions are identified. For example, a single preliminary conclusion is deduced for the Deep Breathing test since the specialists are only interested in the vagal response for this test. In fact, each preliminary conclusion requires a set of input attributes and needs to be classified. In order to identify the input attributes used in this experiment, we spent few weeks in the ANS unit and we have learned how the ANS tests were conducted and the preliminary conclusions were identified by experts. Thus, through several observations and based on the specialists guidelines, the input attributes were identified and used by the C4.5 algorithm.

Table 3 presents all information extracted in order to carry out a classification with C4.5 algorithm. Table 3 was designed by analyzing each test separately and identifying all the necessary attributes based on the empirical knowledge of ANS experts. The attributes Age is common between the different tests which show that it has a great influence on the results interpretation. As an example, in mental stress test, two important values in addition to the age were measured: CSR α and CSR β. Thereafter, two preliminary conclusions are identified in this test: one for the CSR α value, and another for the CSR β value. These conclusions determine whether the CSR α and CSR β values are interpreted as *high*, *normal* or *low;* consequently, two decision trees were generated for mental stress test. For the other tests, one or more decision trees were generated. As a result, eight decision trees were generated and tested. Moreover, the identified classes were coded in order to be used by C4.5 algorithm (see “Generation of the prediction system” section). In fact, to run C4.5 algorithm, three files are required: names file, data file and test file. The data and test files contain the training and test datasets respectively. The names file includes details about the identified classes and attributes: their names and types (discrete or continuous). Thus, the three classes identified for each ANS test were coded and included in the names file as follow: high: 2, normal: 1 and low: 0.

**Table 3:** Details about input data and classes for each ANS tests

ANS tests	Measured values	Input attributes	Class
Deep breathing	Vagal response	Age VR	High Normal Low
Hand grip	Vagal response	Age VR	High Normal Low
	PSR α	Age PSR	High Normal Low
Mental stress test	CSR α	Age CSR α	High Normal Low
	CSR β	Age CSR β	High Normal Low
Orthostatic test	Vagal response	Age VR	High Normal Low
	SP_FC	Age HR_min_ HR_max_	High Normal Low
	SP_TA	Age BP_min_ BP_max_	High Normal Low

### Cardiovascular dysautonomias prediction system: evaluation and validation

The evaluation phase is required to assess the performance of the generated classifiers and the validation phase is adopted to validate the model clinically. In fact, after evaluating our prediction system, a clinical validation is required using the validation set aforementioned in “Medical dataset collection and description” section so as to ensure the efficiency and reliability of the developed system. To do that, a web-based prototype was developed with the Java Enterprise Edition (JEE) platform to enhance visualization and ease of interpretation. JEE provides an API and runtime environment for developing, building and deploying web-based enterprise applications (Jendrock et al. 2014). Thereby, the developed prototype is a web presentation of our prediction system which allows experts to enter the HR and BP values and have as an output the preliminary conclusions generated by the decision trees. As aforementioned, the VR, PSR α, CSR α, CSR β values are calculated using the mathematical Eqs. 1, 2, 3 and 4. Thus, the prototype allows entering just the measured values of HR and BP for the different ANS tests. The mathematical calculations and the preliminary conclusions generation are performed automatically by the prototype.

**Fig. 1:**
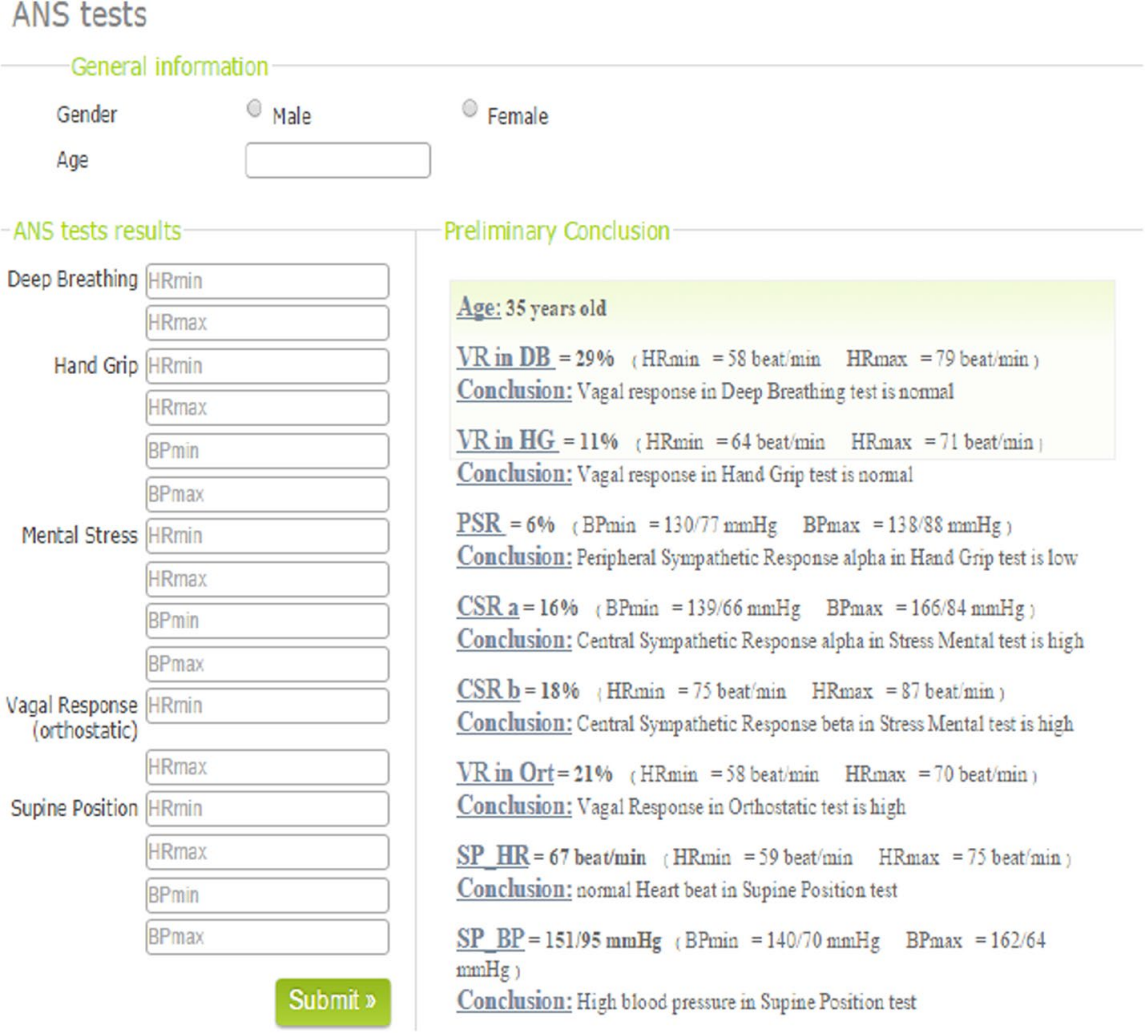
Output of preliminary conclusions for a 35 year-old patient

Figure 1 presents a screenshot of the web based prototype. The screenshot shows the implemented form where input attributes are entered to be processed by the developed prediction system. As shown in Fig. 1, only HR and BP values of ANS tests are required; which will make the analysis of ANS tests results easier for specialists and prevent them from doing all the work manually.

## Results and discussion

### Generation of the prediction system

In order to generate the decision trees, C4.5 algorithm was executed under the Ubuntu distribution of Linux operating system using a C4.5 software release 8. More details about this C4.5 software are available in the following website (www2.cs.uregina.ca, 2015) where the download link and the instructions for use are provided.

The names, data and test files required for the execution of C4.5 algorithm were constructed. Then, the decision trees were generated through several commands. On the other hand, in order to evaluate the performance of the generated classifiers, we used the accuracy metric which is the rate of incorrect classification (Esfandiari et al. 2014). For this reason, the error rate was calculated for each classifier. This metric measures the proportion of errors made over the whole set of instances.

#### Learning phase

**Fig. 2:**
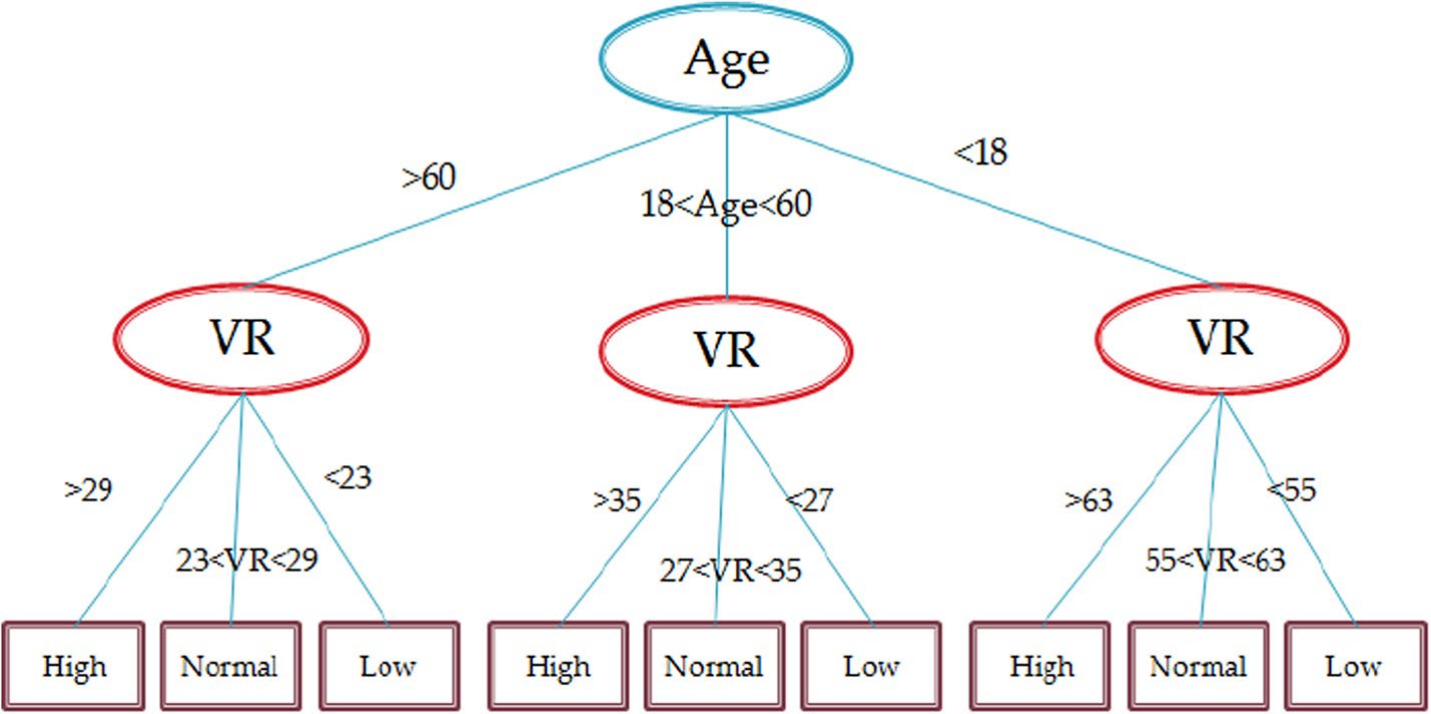
Example of a generated decision tree regarding DB test

In this experimentation, 10 trials were carried out to generate and test the decision trees for each ANS test. In each trial, the training subset as described in “Medical dataset collection and description” section was used to generate the decision tree and calculate its error rate. The training and test subsets were changed in each trial randomly which involves changing data and test files of C4.5 algorithm. Figure 2 presents an example of a generated decision tree of DB test. As previously explained, the age attribute has a great influence on the interpretation of the VR values which is why the age was identified as the root node of the generated decision tree.

Table 4 presents the performance results in terms of error rate on the training set for each ANS test. The values of Table 4 are the mean of error rate values obtained in the 10 trials for each generated decision tree. The mean error rate for each ANS test was calculated using the Eq. 5.

5 $$Mean \,error\, rate = \frac{1}{10}\mathop \sum \limits_{i = 1}^{10} ER_{i}$$

With *ER*_*i*_ = (*TP* + *TN*)/(*TP* + *FP* + *FN* + *TN*) is the error rate of each i^th^ trial. Note that: True positive rate (TP), false positive rate (FP), true negative rate (TN) and false true negative rate (FN) are provided by the used C4.5 software.

**Table 4:** Error rates of the generated classifiers in training set

ANS tests	Phase	Mean error rate (%)
Deep breathing	Vagal response	2.15
Hand grip	Vagal response	3.99
	PSR α	0.81
Mental stress	CSR α	0.85
	CSR β	0
Orthostatic	Vagal response	0
	SP_FC	1.28
	SP_TA	2.54

According to the results of Table 4, the mean values of the error rate are low, which contributes to the increase of the accuracy rate up to 98.54 %. These results may be explained by the fact that input features required for the construction of each decision tree did not include a lot of input attributes. In fact, as shown in “Results and discussion” section, the number of input attributes did not exceed four attributes which help to produce classifiers with high accuracy rates (Quinlan 1993; Han and Kamber 2001).

#### Test phase

The generated decision trees were tested using test sets as aforementioned in “Medical dataset collection and description” and “Learning phase” sections. The error rates measured for these sets were recorded. Table 5 summarizes all the results obtained regarding the test phase and presents the mean value of error rates obtained in the 10 trials for each generated decision tree. The results obtained in the test phase were also encouraging and the values of the error rates recorded were low. Thereby, the classifiers achieved an accuracy rate of 97.76 % for test subset.

**Table 5:** Error rates of the generated classifiers in test set

ANS tests	Phase	Mean error rate (%)
Deep Breathing	Vagal response	0.38
Hand Grip	Vagal response	2.15
	PSR α	3.01
Mental stress	CSR α	0
	CSR β	1.72
Orthostatic	Vagal response	0
	SP_FC	8.96
	SP_TA	1.66

#### Comparison with other classifiers

After evaluating the decision trees generated using test subsets, a comparison between the accuracy rates obtained using C4.5 algorithm, K-NN (Altman 1992) and Naïve Bayes (NB) classifiers (Domingos and Pazzani 1997) was carried out in order to assess the performance of our prediction system. The K-NN and NB classifiers were performed using the Tanagra 1.4 software (www.eric.univ-lyon2.fr, 2015). Table 6 shows the results obtained when applying K-NN and NB classifiers on our data sets. These classifiers were applied on training and test subsets. The mean, max and min values of accuracy rates for each classifier were recorded. In fact, when running the predefined classifiers, several trials were conducted for each ANS test to identify the appropriate neighborhood size for K-NN and the Lambda parameter for Naïve Bayes. Thus, the best results were obtained using a neighborhood size between 3 and 10 for K-NN, and a default Lambda parameter equals to 1.0 for NB. According to Table 6, K-NN achieved an accuracy rate of 97.56 and 93.18 % for training and test sets respectively and Naïve Bayes reached an accuracy rate of 92.73 and 89.79 % for training and test sets respectively. These classifiers have achieved good performance but still lower in comparison with the performance of C4.5 algorithm.

**Table 6:** Comparison of accuracy rates obtained using C4.5, K-NN and Naive Bayes classifiers

Classification techniques	Training sets			Test sets		
	Mean (%)	Min (%)	Max (%)	Mean (%)	Min (%)	Max (%)
C4.5	98.54	96.01	100	97.76	91.04	100
K-NN	96.43	95.12	97.56	85.82	83.33	92.73
Naïve Bayes	89.17	85.25	93.18	85.75	83.82	89.79

### Validation of the prototype cardiovascular dysautonomias prediction system

After assessing the developed prediction system, we have carried out the clinical validation phase using the web prototype. In fact, once the prototype was implemented, it was first approved by the cardiologist responsible of the ANS unit. Then, the prototype was deployed in the ANS unit so as to be evaluated on a set of new patients different from those of the dataset used in the evaluation phase as described in “Medical dataset collection and description” section. The ANS tests results were analyzed and interpreted by the specialists. The preliminary conclusions identified by the specialists were compared to those generated from the prototype. The results of this comparison were analyzed based on three main objectives namely: accuracy, interpretability and usability.

#### Accuracy

Accuracy is one of the most used performance criterion. For classification models, error rate is used as accuracy metric to evaluate their performance. Thereby, in order to measure the error rate of the implemented prototype when validating it in the ANS unit, the preliminary conclusions generated by each decision tree were analyzed separately to determine if they comply with specialists conclusions. The result of each comparison was recorded whether or not there was compliance. This comparison was repeated for each patient record. Eventually, the non-compliance cases recorded in each decision tree were collected to calculate the error rate. Table 7 summarizes the results of the error rates obtained in clinical validation. According to the results presented in Table 7, the error rates recorded in clinical validation are low which contributes to the increase of the accuracy rate up to 99.12 %.

**Table 7:** Error rates of the generated classifiers in clinical validation

ANS tests	Phase	Mean error rate (%)
Deep breathing	Vagal response	0.29
Hand grip	Vagal response	1.89
	PSR α	0.97
Mental stress	CSR α	0.71
	CSR β	1.12
Orthostatic	Vagal response	0.32
	SP_FC	0.67
	SP_TA	1.02

However, as shown in Table 7, there were some cases of non-compliance between the preliminary conclusions generated by the prototype and those identified by the specialists; which can be explained by the fact that some patients were suffering from several dysfunctions at the same time which affects the results interpretation. These exceptions are considered as critical cases and need a deeper analysis by the specialists to produce a correct conclusion. As an example, a 34 year-old patient was suffering from diabetes type 1 for 20 years. She had instable diabetes with unexplained hypoglycemia and hyperglycemia. As a result, the patient was subject of an autonomic dysfunction and underwent the different tests of the ANS units. The results obtained were entered and processed by the implemented prototype. The preliminary conclusions generated by the prototype were compared to those identified by the specialists. These latter were consistent with the conclusions obtained by the prototype except for CSR β phase of mental stress test. In fact, the patient recorded 12 % regarding the central sympathetic response β which is normally interpreted as a normal value for adult patients. However, the specialists interpreted this value as a high central response β which is due to the functional symptoms presented in this case. Thus, a non-compliance case between the conclusions of the prototype and the specialists was recorded.

#### Interpretability

In many domains, interpretability of a prediction system is a fundamental quality characteristic (Vellido et al. 2012). Several experts tend to prefer systems that are more transparent rather than black-box predictive models, because they provide a clear sight on which factors were used to make a particular prediction. Interpretable models can be very convincing, particularly when only a few key factors are used, and each of them is meaningful.

**Fig. 3:**
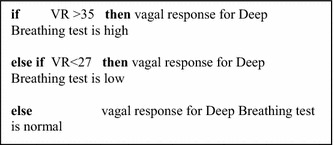
Example of a part the decision tree regarding deep breathing test for a patient who is 35 years old

When we first started developing our decision support system, we aimed to build classification models that are accurate as well as easy to interpret. Thereby, we transformed the decision trees models previously generated to the natural language statements that consists of a series of *if*… *then*… statements where the *if* statements define a partition of a set of features and the *then* statements correspond to the outcome of interest. Using this method, the classification models have become easier to understand and interpret. These models were validated and approved to be interpretable by the experts. Figure 3 presents an example of a part of the decision tree generated regarding Deep breathing test for a patient who is 35 years old using *if*… *then*… statements. Figure 3 shows the different possible interpretation according to VR value for a 35 years old patient which greatly facilitates the deduction of preliminary conclusions. Obviously, this structure differs according to the patient age.

#### Usability

Usability is a quality attribute that assesses how easy user interfaces are to use. It also refers to methods for improving ease-of-use during the design process (Bevan et al. 1991). In other words, usability lies in the interaction of the user with the system and can only be accurately measured by assessing user performance, satisfaction and acceptability. A product is not itself usable or unusable, but a set of metrics are identified to determine the usability for a particular user, task and environment. ISO 9126 defines Usability in terms of five sub-characteristics: Understandability, Learnability, Operability, Attractiveness, and Usability Compliance (Bertoa and Vallecillo 2004). In this study, we assess the usability of the implemented prototype especially learnability metric. Learnability means the capability of the software component to enable the user to learn the application. It enables to assess how long users take to learn how to use particular functions. Seven metrics were identified in ISO 9126 standard to measure the learnability of a developed system. All these metrics aim at evaluating how well the system provides a time saving and a maximum ease of use. In this study, the prototype was designed with the aim to facilitate as much as possible the work of specialists. For this reason, the prototype requires only entering the measured values of HR and BP. The mathematical calculations and the preliminary conclusions are generated automatically and presented as a final result for this stage. Thus, the prototype provides a time saving and ease of use in comparison with the existing method where all the work is done manually.

## Conclusion

In this paper, an experiment about the application of C4.5 decision tree algorithm was conducted using a dataset extracted from the ANS unit of university hospital Avicenne in Morocco. The objective of this study was to produce a decision support system to automate the analysis procedure of the ANS’s test results and make it easier for specialists. In fact, these tests are adopted by specialists to check for signs of autonomic disorder. The results obtained need to be analyzed to deduce preliminary conclusions. These preliminary conclusions are analyzed with other parameters by the specialists to provide a diagnosis of the patient’s state and prescribe the appropriate treatment. Thereby, C4.5 decision tree algorithm was used to define a set of rules helping to generate the preliminary conclusions in the first stage of ANS procedure. The performance of the generated decision trees was measured by calculating error rates values obtained in training and test sets. Thereby, the classifiers of this study achieved an accuracy rate of 98.54 % for training set and 97.76 % for test set respectively. In addition, a comparison between the accuracy rates obtained using C4.5 algorithm, K-NN and Naïve Bayes classifiers was carried out so as to assess the performance of our system. These classifiers have achieved good performance but still lower comparing to the performance reached by C4.5 algorithm.

Moreover, a clinical validation of the cardiovascular dysautonomias prediction system on new patients was carried out. Thereby, JEE platform was adopted to develop the prototype which was deployed in the ANS unit of Avicenne hospital. Several tests were carried out in order to compare the preliminary conclusions provided by the specialists and those generated by our prediction system. The results were analyzed based on three main goals namely: accuracy, interpretability and usability. Hence, the implemented prototype achieved an accuracy rate of 99.12 %. On the other hand, the prediction system can provide decision support for cardiologists to assist them and help them to make better clinical decisions or at least provide them a second opinion. It can also serve as a training tool for nurses and medical students to train them to diagnose patients with cardiovascular dysautonomias.

As a limitation of this system, the size of the dataset used in this research is still quite small. A large dataset would definitely give better results. Moreover, the current cardiovascular dysautonomias prediction system is not yet accomplished. The second stage of the ANS procedure needs to be automated and integrated in the system. In fact, the prototype was deployed in the ANS unit just for validation purposes. Thereby, the system will not be adopted by the specialists until the whole system is completed. For future work, the second stage of the ANS procedure needs to be automated using classification and association techniques. Thereby, a complete cardiovascular dysautonomias prediction system that provide a diagnosis for patients and suggest the appropriate treatment will be produced.
